# Mental Health Intervention for Children with Epilepsy (MICE): cost-effectiveness analysis of psychological therapy in addition to usual care compared with assessment-enhanced usual care alone for children and young people with epilepsy and common mental health disorders

**DOI:** 10.1192/bjo.2025.10916

**Published:** 2025-12-01

**Authors:** Poushali Ganguli, Sophie D. Bennett, Kashfia Chowdhury, J. Helen Cross, Bruce Chorpita, Anna E. Coughtrey, Emma Dalrymple, Peter Fonagy, Tamsin Ford, Isobel Heyman, Rona Moss-Morris, Terence Stephenson, Anaïs d’Oelsnitz, Mariam Shah, James Blackstone, Harriet Quartly, The MICE Consortium, Roz Shafran, Sarah Byford

**Affiliations:** Institute of Psychiatry, Psychology & Neuroscience, King’s College London, London, UK; University College London Great Ormond Street Institute of Child Health, London, UK; Great Ormond Street Hospital for Children NHS Foundation Trust, London, UK; Comprehensive Clinical Trials Unit, University College London, London, UK; Department of Psychology, University of California Los Angeles, Los Angeles, California, USA; Division of Psychology & Language Sciences, University College London, London, UK; Department of Psychiatry, Cambridge University, Cambridge, UK; Cambridge and Peterborough NHS Foundation Trust, Cambridge, UK

**Keywords:** Child and adolescent psychiatry, epidemiology, evidence-based mental health, economic evaluation, childhood epilepsy

## Abstract

**Background:**

Mental health issues are prevalent among children and young people (CYP) with chronic conditions like epilepsy, yet few access evidence-based psychological therapies. Evidence from the Mental Health Intervention for Children with Epilepsy (MICE) trial supports the effectiveness of a personalised modular psychological intervention, but cost-effectiveness is unknown.

**Aims:**

To assess the cost-effectiveness of the MICE intervention compared with assessment-enhanced usual care at 12-months follow-up, taking a health and social care perspective.

**Method:**

We conducted a within-trial economic evaluation. Outcomes were the Strengths and Difficulties Questionnaire (SDQ; primary) and quality-adjusted life years (QALYs; secondary) for CYP, caregivers, and CYP and caregivers combined. Sensitivity analyses examined missing data and intervention-costing assumptions.

**Results:**

Cost-effectiveness results for the SDQ indicated that MICE had a higher probability of being cost-effective compared with control at a willingness to pay ≥£368 per unit improvement. For QALYs, MICE had a lower probability of being cost-effective for CYP compared with control (35 to 42%) across the £20 000–£30 000 per QALY threshold range. However, at the upper threshold this finding was reversed in sensitivity analyses with missing data imputed (45 to 58%) and with MICE costed at 75%, assuming the intervention partly substituted standard services (46 to 55%). Furthermore, MICE had a higher probability of being cost-effective for caregiver QALYs (52 to 63%) and combined CYP and caregiver QALYs (62 to 75%).

**Conclusions:**

MICE appears to be cost-effective compared with assessment-enhanced usual care when considering QALYs for CYP and caregivers combined, though uncertainty exists across willingness-to-pay thresholds.

Mental health difficulties, including depression, anxiety and disruptive behaviours, frequently occur in children and young people (CYP) with chronic conditions like epilepsy. However, a significant number of these individuals do not gain access to evidence-based psychological therapies. Evidence from the Mental Health Intervention for Children with Epilepsy (MICE) randomised controlled trial (RCT) supports the effectiveness of integrating mental health care for CYP within epilepsy services.^1^ The MICE RCT randomised 334 participants (mean age 10.4 years) 1:1 to receive either the MICE intervention (*n* = 166) or assessment-enhanced usual care (*n* = 168). The MICE intervention was a modification of the Modular Approach to Therapy for Children with Anxiety, Depression, Trauma or Conduct problems (MATCH-ADTC), a therapy with demonstrated positive outcomes for CYP^2^ and evidence of a positive cost/benefit ratio^3^ in the USA. Modification involved the addition of content relevant to epilepsy.^4^ At the primary end-point of 6 months, participants receiving the MICE intervention showed significantly lower mean Strengths and Difficulties Questionnaire (SDQ) total difficulties scores compared with the control group (17.6 and 19.6, respectively), with benefits sustained at 12-months follow-up. The trial demonstrated that mental health comorbidities in CYP with epilepsy can be effectively and safely treated by a variety of clinicians using an integrated intervention model.

While the MICE intervention adds to the cost of supporting CYP, its integration within epilepsy clinics and delivery by clinicians with limited prior experience in psychological therapy might incur lower costs than contact with child and adolescent mental health services (CAMHS) and may reduce the need for additional services over time. A systematic review identified several economic evaluations of psychological interventions for adults with physical health conditions,^5^ but none focused on epilepsy. For CYP, a systematic review of the effectiveness of brief psychological interventions for CYP with long-term physical health conditions concluded that there was ‘insufficient evidence to assess whether these interventions are cost-effective’.^6^

This study presents the findings of an economic evaluation conducted as part of the MICE trial,^1,7^ which aimed to assess the cost-effectiveness of adding the MICE intervention to usual care (MICE group) compared with assessment-enhanced usual care alone (control group) for CYP with epilepsy and common mental health disorders at 12-months post-randomisation.

## Method

### Study design, setting and procedures

Detailed information on the design, methods and procedures of the MICE RCT are reported elsewhere.^1,7^ In brief, the MICE trial was a multi-centre, parallel group, superiority RCT to evaluate the efficacy of the MICE intervention delivered within 13 epilepsy services for CYP with epilepsy and common mental health difficulties (anxiety, depression or disruptive behaviour) in England and Northern Ireland. The trial was approved by the South Central - Oxford Research Ethics Committee (18/SC/0250) and prospectively registered (ISRCTN57823197). The authors assert that all procedures contributing to this work comply with the ethical standards of the relevant national and institutional committees on human experimentation and with the Helsinki Declaration of 1975, as revised in 2013.

### Participants

Participants were randomised between August 2019 and February 2022 using a 1:1 ratio through a web-based system (https://www.sealedenvelope.com). Randomisation included the following minimisation factors: primary mental disorder (anxiety, depression or disruptive behaviour), presence of autism spectrum disorder diagnosis as reported by the caregiver (yes, no), age (<11, ≥11 years) and presence of intellectual disability as reported by the caregiver (yes, no). Trial participants, caregivers and clinicians were not blind to treatment allocation. Researchers blind to treatment allocation carried out outcome assessments at 6- and 12-month follow-up.

Participants were eligible for inclusion if they: were attending UK National Health Service (NHS) epilepsy clinics; were aged between 3 and 18 years; scored above the pre-specified threshold on the SDQ^8,9^ (total difficulty score ≥14 and impact score≥2); met Development and Wellbeing Assessment (DAWBA)^10^ DSM-5^11^ diagnostic criteria for a mental disorder and had a parent/caregiver willing to participate in the trial. Exclusion criteria are reported in the supplementary material available at https://doi.org/10.1192/bjo.2025.10916. Written informed consent was provided by parents/caregivers, and consent or assent was provided by CYP for cases in which this was appropriate.

### Intervention

The MICE intervention was a modified form of MATCH-ADTC, which is a personalised modular cognitive–behavioural intervention with content relevant to epilepsy integrated throughout. In addition, it contained a compulsory epilepsy-specific module and three epilepsy-related optional modules.^12^ The intervention included an initial assessment session followed by weekly intervention sessions delivered via telephone or video conferencing, although in-person sessions were supported if they were strongly preferred by the family or clinically indicated. The therapy included up to 20 sessions, delivered within 6-months of randomisation, plus two booster sessions.

Usual care varied and typically included referral to hospital-based paediatric psychology services or to CAMHS. Usual care was ‘assessment-enhanced’ as it included providing detailed diagnostic results from the DAWBA to the general practitioner (GP), other clinical team members and the caregiver.

### Economic perspective

The economic evaluation took the NHS and personal social services perspective preferred by the National Institute for Health and Care Excellence (NICE),^13^ and included participant use of all health and social care services.

### Method of economic evaluation

While cost-utility analysis, which uses quality adjusted life years (QALYs) estimated from self-reported health-related quality of life (HRQoL) data, is preferred by NICE,^13^ there are currently no tools suitable for self-reported measurement of HRQoL in young children (those under 7). As a result, a proxy report is required which reduces the validity of the responses. For this reason, the pre-specified primary economic evaluation was a cost-effectiveness analysis at 12-months post-randomisation, explored using the primary clinical measure of outcome (parent-reported SDQ total difficulties score).^9^ Pre-specified secondary economic analyses considered: (a) cost-effectiveness analysis using SDQ at 6-months post-randomisation (primary clinical end-point)^1^; (b) cost-utility analysis focused on CYP using QALYs calculated from the Child Health Utility (CHU9D) measure of HRQoL, using a proxy report where necessary^14^; (c) cost-utility analysis focused on caregivers using QALYs calculated from the EQ-5D-5L measure of HRQoL^15,16^; and (d) cost-utility analysis using QALYs for CYP and caregivers combined.

### Service use and cost

Health and social care service use was recorded in telephone interviews using the Child and Adolescent Service Use Schedule (CA-SUS), a service use measure tailored for mental health populations and adapted to include epilepsy-relevant services.^12,17,18^ Data were collected from caregivers at baseline (which covered the previous 3 months) and at 6- and 12-months post-randomisation (covering the period since last assessment). Data on the use of the MICE intervention and training and supervision for MICE therapists were obtained from trial records.

Nationally applicable unit costs were applied to service use items reported in the CA-SUS in order to calculate total costs per participant (Supplementary Table S1). Unit costs were in UK pounds sterling for the 2020/21 financial year. Discounting of costs was not needed as follow-up was less than one year. The cost of the MICE intervention (Supplementary Table S2) was calculated using recommended micro-costing approaches and based on NHS Agenda for Change Band 5 therapists delivering the intervention, with supervision provided by Band 7 and Band 8a clinical staff. Staff costs were based on published costs per working hour,^19^ which includes salaries, on-costs (e.g. national insurance and superannuation paid by the employer) and relevant overheads (e.g. administrative, capital and managerial). Costs were adjusted to account for the time therapists spent on indirect activities (preparation, supervision, administration, etc.) by applying a ratio of face-to-face to non-face-to-face activities estimated from questionnaires completed by MICE trial therapists and the costs of training and supervision were added.

### Outcomes

Outcome measures (SDQ, CHU9D and EQ-5D-5L) were reported at baseline, 6- and 12-months post-randomisation. The SDQ assesses behaviours, emotions and peer relationships in CYP, with scores ranging from 0 to 40, with higher scores indicating higher symptoms of a mental health condition.^9^ The CHU9D includes nine dimensions (worried, sad, pain, tired, annoyed, schoolwork, sleep, daily routine and usual activities) while the EQ-5D-5L includes five (mobility, self-care, usual activities, pain/discomfort and anxiety/depression). In both measures, dimensions are scored on five levels to indicate severity. Health states based on item scores were converted into a summary utility score through the application of quality weights derived from the valuation of each measure in UK general population samples.^20,21^ Utility scores vary between 0 (dead) and 1 (perfect health) and indicate population preferences for individual health states. QALYs were then calculated as the area under the curve which is defined by the utility scores at baseline and the 6- and 12-month follow-ups,^22^ assuming linear change between measurement time-points. Measures were completed using the following approaches:

#### CYP

- Child self-report version of the CHU9D, completed by CYP aged 7 years and over who were judged by research assessors to be able to complete the measure, taking intellectual disabilities into account.
- Caregiver proxy-report version of the CHU9D for children over 5 years old, completed by caregivers of CYP aged 5 years or over.
- Caregiver proxy-report version of the CHU9D for children under 5 years old, completed by caregivers of children under 5 years old.

#### Caregivers

- EQ-5D-5L, completed by caregivers.

All caregivers proxy-reported the relevant version of the CHU9D on behalf of their child. Preference was given to self-report data, with a caregiver report used where self-report was not feasible. However, in practice, few CYP self-reported (approximately 1% of the total sample), so the data is almost entirely caregiver reported.

### Data analysis

Service use data are presented by group as the mean, s.d., range and percentage who had at least one contact. Total costs and outcomes are summarised by group using the mean and s.d. Differences in mean costs were analysed using parametric *t*-tests. While cost data are often skewed, the use of parametric tests is recommended to enable inferences to be made about the arithmetic mean, a more meaningful statistic for cost data.^23^

Cost-effectiveness was assessed in terms of incremental cost-effectiveness ratios (the additional cost divided by additional benefit of intervention versus control) and used the net benefit approach.^24^ Mixed effects linear regression models were used to estimate the mean net monetary benefit between intervention groups. A joint distribution of incremental mean costs and effects was generated using non-parametric bootstrapping to explore the probability that each intervention is the optimal choice, subject to a range of possible maximum values (ceiling ratio) that decision-makers might be willing to pay for a unit improvement in outcomes. Uncertainty around the estimates of cost and effectiveness was presented by plotting these probabilities for a range of values of the ceiling ratio on cost-effectiveness acceptability curves, which illustrate the probability that an intervention is cost-effective compared with a comparator for a range of possible ceiling ratio values.^25^ While there is no universally acknowledged willingness-to-pay threshold for the SDQ, for QALYs an indicative threshold of £20 000 to £30 000 per QALY is applied by NICE. All analyses were adjusted for baseline variables of interest (cost, SDQ and QALY) and minimisation factors (mental disorder, age, presence of autism spectrum disorder or presence of intellectual disability).

Pre-specified sensitivity analysis examined the impact of missing data by using multiple imputation with chained equations.^26^ Thirty-one imputed data-sets were generated using predictive mean-matching for costs, QALYs and SDQ scores at each time-point and combined using Rubin’s rules.^27^ The imputation model included the outcomes of interest (costs, QALYs and SDQ score), trial group status and all minimisation factors listed above. A post-hoc sensitivity analysis assessed assumptions made in costing the MICE intervention. MICE sessions were initially assumed to be additional to standard services provided within epilepsy clinics, with new staff being employed to deliver the intervention. However, in practice, two aspects of intervention delivery during the trial challenged this assumption. First, while some clinics recruited an additional staff member, others trained existing staff. Second, some therapists reported providing the intervention as a substitute for standard services, which typically includes some psychosocial support. Delivery by existing staff and as a substitute for standard care, reduces the cost of an intervention compared with a service that is wholly additional to standard care. Consequently, a sensitivity analysis was undertaken applying 75% of the full cost of the intervention. This proportion was based on discussions with the MICE research team and assumed that 50% of services would employ new staff, while 50% would train existing staff and 50% of the intervention delivered by existing staff would substitute for standard care.

## Results

### Participants and data completeness

A total of 334 participants were eligible for inclusion and randomised to MICE (*n* = 166) or control (*n* = 168). Full participant details are reported in the clinical paper^1^ and baseline characteristics summarised in Supplementary Table S3. Availability of economic data is summarised in Supplementary Table S4. For the SDQ, economic data were 84 and 78% complete at 6- and 12-months, respectively. For QALYs, completeness ranged from 65 to 66%, dependent on the analysis.

### Service use

Health and social service use over 12-month follow-up is reported in Supplementary Table S5. Services used most frequently included out-patient appointments for epilepsy (approximately 85% of participants), out-patient appointments for ‘other’ non-mental health reasons (approximately 70%) and community contact with nurses (approximately 73%). There was also substantial use (>20% of participants) of in-patient admissions for epilepsy and ‘other’ non-mental health reasons, contact with accident and emergency, ambulance services, GP, community paediatricians, CAMHS, speech and language, occupational and physiotherapists, and educational psychologists. The proportion of participants using services was generally lower in the MICE group compared with control, notably for in-patient admissions for ‘other’ reasons (13% MICE, 25% control), out-patient contacts for ‘other’ reasons (68% MICE, 74% control), and ambulance services (14% MICE, 28% control). Mean use of services (e.g. number of contacts, nights, etc.) was also lower in the MICE group (Supplementary Table S5).

Focusing on mental health services, use was generally lower in the MICE group. For example, out-patient contacts for mental health (12% MICE, 14% control), community psychiatrists (2% MICE, 11% control), clinical psychologists (10% MICE, 17% control) and counselling (3% MICE, 12% control). Mean use of these services was also lower in the MICE group (Supplementary Table S5). While contact with CAMHS was slightly higher in the MICE group (20% MICE, 18% control), the mean number of CAMHS contacts was lower (mean 0.77 MICE, 0.86 control).

The percentages of CYP prescribed medications were similar between groups (Supplementary Table S6). Over 90% of participants were prescribed medication for epilepsy (96% MICE, 92% control) and approximately half were prescribed medications for ‘other’ non-mental health reasons (48% MICE, 46% control). Medications for mental health conditions were prescribed infrequently (6% MICE, 8% control) and these were commonly for depression, anxiety or attention-deficit hyperactivity disorder.

### Costs

Total costs over 12-months follow-up are reported in Table 1. With the exception of community health and social services, the MICE group incurred lower costs than the control group in all categories, although differences were not statistically significant. Total costs excluding the MICE intervention were £6421 in the MICE group, compared with £7845 in the assessment-enhanced usual care group, an observed difference of £1423 and adjusted difference of £796 (95% CI −2628 to 1037; *p* = 0.395).

**Table 1:** Costs over the 12-month follow-up period (£)

Cost category	Intervention (*N* = 128)		Control (*N* = 133)		Unadjusted difference	Adjusted difference	95% CI	*p*-value
	Mean	s.d.	Mean	s.d.				
Hospital services	3134.44	3707.49	4617.08	7007.13	−1482.64	−783.36	−1917.51 to 350.79	0.176
Community services	1870.87	3470.70	1784.14	2828.13	86.72	130.29	−577.94 to 838.53	0.718
Accommodation	244.45	1936.58	259.83	1303.77	−15.39	−141.67	−440.50 to 157.17	0.353
Medication	1171.65	3633.18	1183.46	5079.90	−11.80	−21.58	−1082.45 to 1039.29	0.968
Total excluding MICE	6421.40	6586.18	7844.51	10349.54	−1423.11	−795.53	−2628.44 to 1037.38	0.395
Intervention	1466.23	313.22	NA	NA	NA	NA	NA	NA
Total including MICE	7887.63	6595.01	7844.51	10349.54	43.12	668.61	−1166.39 to 2503.61	0.475

MICE, Mental Health Intervention for Children withEpilepsy; NA, not applicable.

The cost of the MICE intervention was estimated to be £80.41 per session (Supplementary Table S2) and a total of £1466.23 per participant, on average, which is relatively high compared with other psychological interventions which typically have fewer sessions than the MICE intervention (mean MICE sessions 18.23, range 2 to 23 sessions). When added to the cost of all other health and social services, the cost difference between the groups was negligible (£7888 MICE, £7845 control; observed mean difference £43). However, the adjusted mean difference was higher for MICE compared with control, although not statistically significant (adjusted mean difference £668.61; 95% CI −1166 to 2504; *p* = 0.475). This was primarily due to lower baseline costs in the MICE group compared with the assessment-enhanced usual care group (£1602 MICE, £2076 control; Supplementary Table S3).

### Outcomes

Table 2 presents SDQ total difficulties scores and QALYs at 12-months follow-up. SDQ (adjusted mean difference −2.021; 95% CI −3.199 to 0.844; *p* < 0.001), QALYs for CYP (adjusted mean difference 0.023; 95% CI 0.002 to 0.044; *p* = 0.031) and QALYs for CYP plus caregivers (adjusted mean difference 0.093; 95% CI 0.021 to 0.164; *p* = 0.010) were all statistically significantly better in the MICE group compared with assessment-enhanced usual care. These findings align with the clinical results, indicating a significant effectiveness advantage for MICE compared with control.^1^

**Table 2:** Outcomes at the 12-month follow-up point

Outcome measure	Intervention (*N* = 128)		Control (*N* = 133)		Unadjusted difference	Adjusted difference	95% CI	*p*-value
	Mean	s.d.	Mean	s.d.				
SDQ^a^	17.039	6.410	19.624	6.570	−2.585	−2.021	−3.199 to −0.844	<0.001
QALYs^b^ (child)	0.811	0.092	0.773	0.115	0.038	0.023	0.002 to 0.044	0.031
QALYs^b^ (caregiver)	0.809	0.164	0.764	0.234	0.046	0.036	−0.003 to 0.075	0.076
QALYs^b^ (combined)	1.619	0.220	1.537	0.318	0.082	0.093	0.021 to 0.164	0.010

SDQ, Strengths and Difficulties Questionnaire total score; QALY, quality adjusted life year.

a. Lower score, better outcome.

b. Higher score, better outcome.

### Cost-effectiveness

**Fig. 1:**
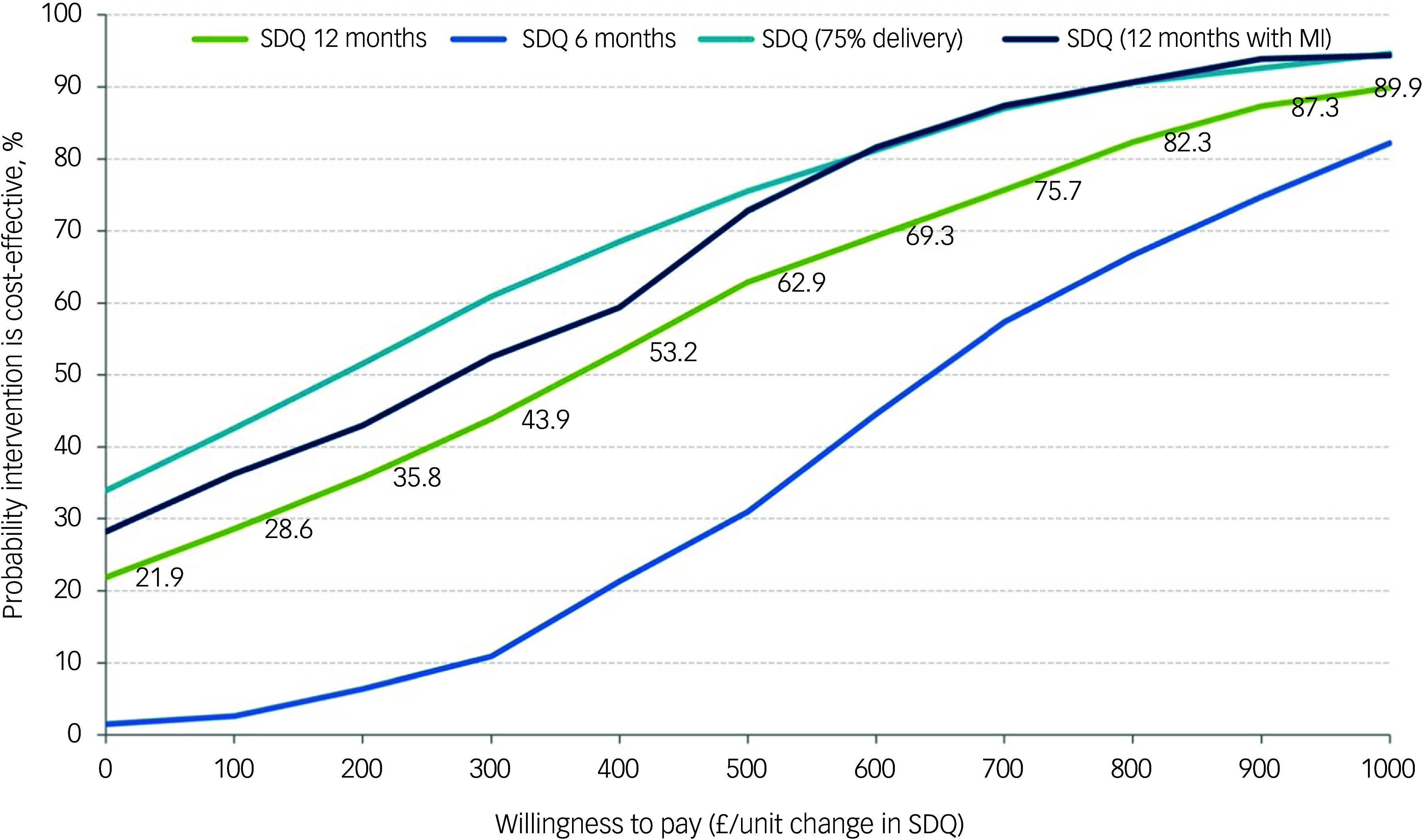
Cost-effectiveness acceptability curves for Strengths and Difficulties Questionnaire (SDQ). MI, Mental Health Intervention for Children with Epilepsy intervention.

**Fig. 2:**
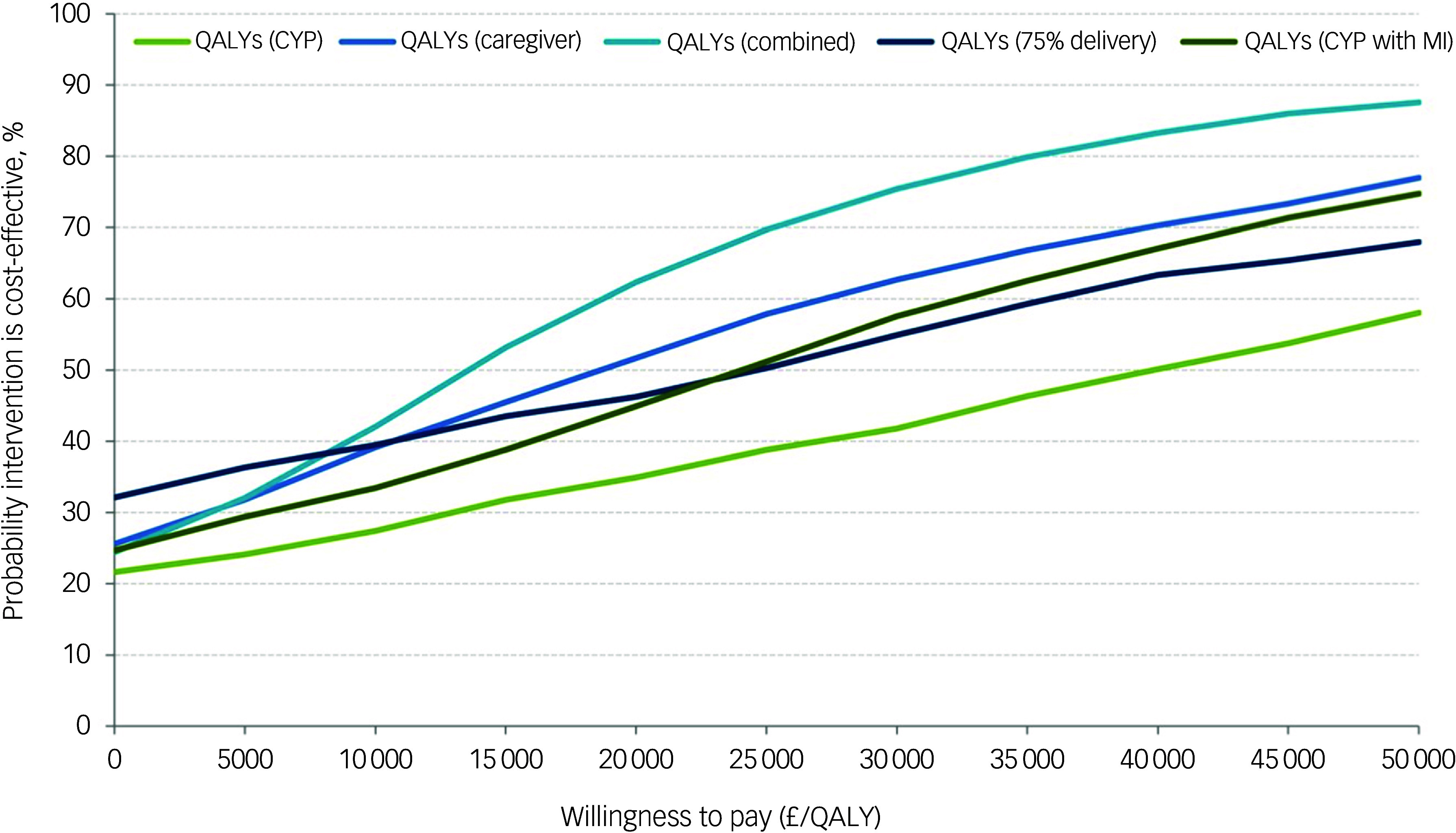
Cost-effectiveness acceptability curves for quality adjusted life years (QALYs). CYP, children and young people; MI, Mental Health Intervention for Children with Epilepsy intervention.

All incremental cost-effectiveness ratios, presented in Table 3, involved a trade-off, with the MICE group generating better outcomes for higher costs than the control group. This is illustrated in the cost-effectiveness planes for SDQ and QALYs (Supplementary Figures S1 and S2, respectively), which show the bootstrapped replications falling predominantly to the right of the y-axis (higher effectiveness MICE versus control) and above the *x*-axis (higher costs MICE versus control). Cost-effectiveness acceptability curves for the SDQ (Fig. 1) suggest there was a low probability of MICE being cost-effective for low values of willingness-to-pay for improvements in SDQ but a high probability as willingness-to-pay increases. The point at which the MICE intervention had a higher probability of being cost-effective than the control (above 50%) was at a willingness-to-pay of £368 per unit improvement in SDQ at 12-month follow-up, £640 at 6-month follow-up and £184 for the sensitivity analysis with 75% intervention cost. For CYP QALYs (Fig. 2), the probability of MICE being cost-effective compared with the control was 35 to 42% across the NICE threshold range of £20 000 to £30 000 per QALY in complete case analysis but increased to 45 to 58% with missing data imputed. MICE also had a higher probability of being cost-effective compared with the control for all other QALY analyses: 52 to 63% for caregivers alone, 62 to 75% for CYP and caregivers combined and 45 to 55% with 75% intervention cost.

**Table 3:** Incremental cost-effectiveness ratios (ICERs) for primary, secondary and sensitivity analyses

Analysis	*n*	Mean difference in cost, £	Mean difference in outcome	ICER, £	Probability cost-effective, %
Primary analysis					
SDQ at 12-months^a^	261	660.15	−2.021	−326.58 per unit SDQ	21.9 to 89.9
Secondary analyses					
SDQ at 6-months^a^	279	1277.84	−2.011	−635.36 per unit SDQ	1.5 to 82.2
QALYs at 12-months for CYP^b^	220	696.63	0.022	31 341.46 per QALY	34.9 to 41.8
QALYs at 12-months for caregivers^b^	220	593.04	0.036	16 627.65 per QALY	51.7 to 62.7
QALYs at 12-months combined^b^	218	569.64	0.090	6311.15 per QALY	62.4 to 75.4
Sensitivity analyses using multiple imputation					
SDQ at 12-months^a^	334	469.64	−2.006	−234.07 per unit SDQ	28.2 to 94.4
QALYs at 12-months for CYP^b^	334	493.23	0.022	22 504.77 per QALY	44.9 to 57.6
Sensitivity analyses using 75% intervention cost					
SDQ at 12-months^a^	261	305.50	−2.021	−151.13 per unit SDQ	33.9 to 94.6
QALYs at 12-months for CYP^b^	220	343.41	0.022	15 449.88 per QALY	46.2 to 54.9

SDQ, Strengths and Difficulties Questionnaire; QALYs, quality adjusted life years; CYP, children and young people.

a. Based on a willingness-to-pay for a one-point improvement in SDQ score between £0 and £1000.

b. Based on a willingness-to-pay for a QALY between £20 000 and £30 000.

## Discussion

### Summary of findings

In this study, we present evidence to suggest that the MICE modular mental health intervention for CYP with epilepsy and mental health difficulties has a higher probability of being cost-effective than assessment-enhanced usual care when considering QALYs for: (a) both CYP and caregivers and (b) caregivers alone across the £20 000 to £30 000 per QALY range; and at the upper threshold of £30 000 per QALY for: (c) CYP alone adjusted for missing data and (d) CYP alone when intervention costs were adjusted assuming the intervention is not wholly additional to standard epilepsy clinic services. The primary economic evaluation focused on the SDQ was inconclusive due to the lack of a recognised willingness-to-pay for unit changes in the SDQ score.

The primary analysis costed the MICE intervention as a distinct and supplementary service to services typically accessed by CYP in epilepsy clinics. However, there was evidence to suggest that the intervention replaced some level of standard care provided by epilepsy clinics and adjustment of the cost of the intervention to acknowledge this substitution effect altered the results focused on CYP alone, such that there was a higher probability of the MICE intervention being cost-effective compared with the control. The extent of substitution is uncertain, and assumptions made were speculative, but the results suggest that the cost-effectiveness of MICE can be enhanced through training of existing staff to deliver the MICE intervention as part of usual care in epilepsy services, rather than hiring new staff.

The probability of the MICE intervention being cost-effective compared with assessment-enhanced usual care was highest when QALYs were combined for CYP and their caregivers. This is an important finding, given evidence to suggest that parents – particularly mothers – of children with epilepsy are at increased risk of mental health difficulties and poorer quality of life than healthy controls.^28,29^ In the present study, over 90% of caregivers were female in both trial groups. Funding decisions should therefore take the impact of the MICE intervention on caregivers into consideration, in addition to the impact for CYP. Future research should consider the impact on the wider family, particularly siblings whose own mental health might be adversely impacted by their sibling’s health conditions.^30,31^

Use of mental health services was generally low, despite participants meeting SDQ criteria for mental health symptoms and diagnostic criteria for a mental health disorder. Only 18% of the control group reported any contact with CAMHS over the 12-month follow-up, suggesting significant unmet mental health need. The MICE intervention is a lower cost per contact than CAMHS and is likely to be more acceptable and less disruptive to families, since it is provided as part of routine contact with epilepsy services. In addition, qualitative interviews have found it to be acceptable to health professionals trained to deliver the intervention in participating epilepsy clinics.^32^ Given current capacity constraints in CAMHS, the MICE intervention has the potential to be a lower cost, more acceptable and more accessible alternative to referral to CAMHS.

It is important to note that participants in the control group received assessment-enhanced usual care, where comprehensive mental health assessment results were shared with caregivers and clinical teams. The control group showed some improvement and increased service use compared with the MICE group, suggesting the assessment component itself may have initiated service use and clinical benefits. In a comparison against standard usual care alone, control group costs would likely have lower service utilisation but potentially poorer clinical outcomes, suggesting that the present cost-effectiveness estimates may be conservative.

The MICE intervention benefited from building upon an existing evidence-based therapy for CYP with anxiety, depression, trauma or conduct disorder (MATCH-ADTC) which greatly reduced the development costs of the MICE therapy. Future roll-out of the intervention might additionally benefit from lower training costs than those estimated in the current study, as small-group training and supervision from programme developers are typically more costly than wider implementation involving a process of ‘training the trainers’, with embedded trainers providing ongoing training to new professionals with minimal involvement of the developer team.

### Strengths and limitations

This study benefited from integration within a robust multi-centre RCT, achieving high follow-up rates, and broad inclusion of CYP, including those with intellectual disabilities. The economic component was limited by the absence of an outcome measure suitable for young children that facilitates economic evaluation. Although the SDQ is recognised as valid and reliable for CYP, the absence of an agreed willingness-to-pay threshold for the SDQ rendered the results inconclusive. In contrast, QALYs facilitate decision-making through willingness-to-pay thresholds preferred by NICE, but the absence of an agreed, robust method of measurement of HRQoL in young children (those under 7) and in older children with intellectual disabilities, precludes self-report. To address this limitation, we used proxy-reporting by parents, which supported cost-utility analysis using QALYs. Multiple imputation had a greater impact on cost estimates than QALY estimates, reflecting the higher variability and skewed distribution of healthcare costs compared with the more constrained range for QALYs. Finally, there is no standardised or accepted approach to combining QALYs from different populations (CYP and adults) or when estimated using different measures (CHU9D and EQ-5D-5L).

This study was conducted during the COVID-19 pandemic, which significantly disrupted UK healthcare delivery patterns through cancelled appointments, lockdowns and transitions to remote consultations. While these did have an impact on service use, these impacts were universal across the healthcare system and thus unlikely to have had a differential effect by trial group. Therefore, while the absolute costs observed may not reflect typical healthcare utilisation patterns, the findings regarding the relative cost-effectiveness of the MICE intervention compared with assessment-enhanced usual care remain robust.

### Implications

Implementation of the MICE intervention within routine epilepsy services demonstrates potential cost-effectiveness compared with assessment-enhanced usual care for CYP with epilepsy and coexisting common mental health disorders when considering the impact on the HRQoL of CYP and their parents/caregivers combined. Most analyses demonstrated probabilities of cost-effectiveness exceeding 50% at the upper NICE threshold of £30 000 per QALY, though probabilities were generally lower at the £20 000 per QALY threshold, highlighting both the importance of threshold selection in decision-making and the need for careful consideration of economic evidence alongside clinical benefits. MICE may also be cost-effective compared with assessment-enhanced usual care for CYP alone, if intervention delivery substitutes for a proportion of standard care within epilepsy services, as happened in some services during the trial.

The control group in this study received assessment-enhanced usual care, which appeared to increase mental health service use compared with the MICE group, suggesting that cost-effectiveness estimates may be conservative compared with standard usual care without enhanced assessment. Future research should investigate the cost-effectiveness of comprehensive mental health assessment alone and consider the potential for further adaptation of the MATCH-ADTC intervention and integration within services supporting other chronic conditions in CYP.

## Supporting information

Ganguli et al. supplementary material Ganguli et al. supplementary material

## Data Availability

Data are not publicly available. All requests for data will be reviewed by the Mental Health Intervention for Children with Epilepsy (MICE) study team, to verify whether the request is subject to any intellectual property or confidentiality obligations. Requests for access to the participant-level data from this study can be submitted via email to the corresponding author with detailed proposals for approval. A signed data access agreement with the MICE team is required before accessing shared data. Code is not made available as we have not used custom code or algorithms central to our conclusions.

## Supplementary material

The supplementary material is available online at https://doi.org/10.1192/bjo.2025.10916
