# Supplementary material for: Mental Health Intervention for Children with Epilepsy (MICE): cost-effectiveness analysis of psychological therapy in addition to usual care compared with assessment-enhanced usual care alone for children and young people with epilepsy and common mental health disorders
Source: BJPsych Open. 2025 Dec 1;12(1):e4. doi: 10.1192/bjo.2025.10916 (PMC12724111; doi:10.1192/bjo.2025.10916)
Supplement: Ganguli et al. supplementary material [file S2056472425109162sup001.docx]

# **Mental Health Intervention for Children with Epilepsy (MICE): Cost-effectiveness analysis of psychological therapy in addition to usual care compared to assessment-enhanced usual care alone for children and young people with epilepsy and common mental health disorders**

**Supplement**

**Exclusion criteria**

Exclusion criteria included: not speaking/understanding English well enough to complete screening assessments; having an intellectual disability at a level that precluded access to the measures and/or intervention; screening results indicating a severe mental health disorder not considered suitable for the trial intervention; actively receiving, or due to receive during the study period, intensive psychological input focused on cognitive and/or behavioural strategies to address emotional or behavioural difficulties; refusing to consent to the research team contacting their GP/other relevant health professionals about their participation in the research; refusing to have the trial therapy sessions audio and/or video recorded; unable to complete the screening measures (SDQ and DAWBA).

**Unit costs**

Unit costs are summarised in Table S1. Unit costs for hospital contacts were obtained from NHS reference costs.^1^ Unit costs for community-based services, as well as accommodation provided by Local Authorities, were obtained from an annual compendium of unit costs of health and social care.^2^ The costs of medications were based on prices listed in the British National Formulary for Children^3^ and Prescription Cost Analysis data (NHS Business Services Authority Statistics).^4^

**Table S1: Unit costs**

| **Service** | **Unit cost (£)** | **Source** |
| --- | --- | --- |
| **Hospital services** |  |  |
| Inpatient - Epilepsy | 922.55 | 1 |
| Inpatient - Mental health | 1066.25 | 1 |
| Inpatient - Other | 1159.48 | 1 |
| Outpatient - Epilepsy | 308.00 | 1 |
| Outpatient - Mental health | 373.60 | 1 |
| Outpatient - Other | 258.00 | 1 |
| Accident emergency | 188.77 | 1 |
| Ambulance | 292.09 | 1 |
| **Community services** |  |  |
| General practitioner | 39.23 | 2 |
| Nurse | 21.00 | 2 |
| Community paediatrician | 76.45 | 1 |
| Psychiatrist | 131.74 | 1 |
| CAMHS | 254.90 | 1 |
| Clinical psychologist | 55.57 | 1 |
| Counsellor | 51.00 | 1 |
| Art therapy | 90.00 | 2 |
| Occupational therapy | 93.00 | 2 |
| Family therapy | 93.00 | 2 |
| Dietician | 92.00 | 2 |
| Social worker | 23.00 | 2 |
| Youth worker | 23.00 | 2 |
| Family support worker | 23.00 | 2 |
| Inclusion worker | 23.00 | 2 |
| Education psychologist | 51.00 | 2 |
| Speech and language therapy | 84.00 | 2 |
| Physiotherapist | 94.00 | 2 |
| **Local authority accommodation** |  |  |
| Foster care | 94.43 | 2 |
| Residential care | 721.00 | 2 |
| Respite care | 467.00 | 2 |

CAMHS=Child and Adolescent Mental Health Services

1 National Cost Collection: National Schedule of NHS costs, 2019-20

2 Personal Social Services Research Unit (PSSRU) Unit Costs of Health and Social Care 2021

**Table S2: Intervention cost**

| **Cost for** | **Cost per session** | **Source** | **Notes** |
| --- | --- | --- | --- |
| Delivery | 77.90 | PSSRU | Based on cost per working hour of Band 5 clinical staff and direct to indirect ratio of 0.9 |
| Supervision* | 2.04 | PSSRU | Based on average cost per working hour of Band 7 and Band 8a clinical staff, and on assumption that supervisors provide three hours of supervision each week during period of intervention delivery |
| Training* | 0.47 | Trial data | Based on costs of initial training (five-day face-to-face workshop delivered to groups of 1-4 health professionals (minimum NHS Band 5/graduate level)) and ongoing consultations with developers of the intervention, and on assumption that training is valid for three years and therapists deliver the same number of sessions each year |
| Total | 80.41 |  |  |

PSSRU=Personal Social Services Research Unit: Unit Costs of Health and Social Care 2021

More detailed description of the training and supervision approach is provided in Coughtrey et al. (2024)^5^

**Table S3: Baseline characteristics**

| **Participant characteristics** | **MICE**  **(N=166)** | **Control**  **(N=168)** |
| --- | --- | --- |
| Age (years); mean (SD) | 10.5 (3.6) | 10.3 (4.0) |
| Age (years); n (%) |  |  |
| <11 | 99 (59.6) | 101 (60.1) |
| 11 or more | 67 (40.4) | 67 (39.9) |
| Gender; n (%) |  |  |
| Female | 81 (48.8) | 87 (51.8) |
| Male | 85 (51.2) | 81 (48.2) |
| Ethnicity; n (%) |  |  |
| White or White British | 122 (73.5) | 116 (69.0) |
| Mixed | 18 (10.8) | 18 (10.7) |
| Asian or Asian British | 11 (6.6) | 12 (7.1) |
| Black or Black British | 7 (4.2) | 16 (9.5) |
| Other ethnic groups | 4 (2.4) | 5 (3.0) |
| Did not disclose | 4 (2.4) | 1 (0.6) |
| Socio economic status; n (%) |  |  |
| Most deprived 20% | 17 (11.0) | 23 (14.4) |
| 20 - 40% | 42 (27.1) | 40 (25.0) |
| 40 - 60% | 38 (24.5) | 40 (25.0) |
| 60 - 80% | 20 (12.9) | 24 (15.0) |
| Least deprived 20% | 38 (24.5) | 33 (20.6) |
| Primary mental health disorder; n (%) |  |  |
| Anxiety | 66 (39.8) | 67 (39.9) |
| Depression | 7 (4.2) | 9 (5.4) |
| Disruptive behaviour | 93 (56.0) | 92 (54.8) |
| SDQ Total Difficulties; mean (SD) | 23.0 (5.2) | 23.5 (5.4) |
| CHU9D; mean (SD) | 0.80 (0.12) | 0.78 (0.13) |
| Caregiver EQ-5D-5L; mean (SD) | 0.80 (0.22) | 0.78 (0.25) |
| Costs 3-months prior to trial entry; mean (SD) | 1601.84 (2200.70) | 2075.98 (3124.56) |

**Table S4: Data completeness for the economic evaluation**

|  | **MICE**  **N (%)** | **Control**  **N (%)** |
| --- | --- | --- |
| **Baseline** |  |  |
| Service use | 166 (100%) | 168 (100% |
| SDQ | 166 (100%) | 168 (100%) |
| CHU9D | 166 (100%) | 168 (100%) |
| EQ-5D-5L | 166 (100%) | 168 (100%) |
| **6-month follow-up** |  |  |
| Service use | 136 (82%) | 144 (86%) |
| SDQ | 160 (96%) | 158 (94%) |
| CHU9D | 131 (79%) | 126 (75%) |
| EQ-5D-5L | 130 (78%) | 126 (75%) |
| **12-month follow-up** |  |  |
| Service use | 138 (83%) | 139 (82%) |
| SDQ | 153 (92%) | 153 (91%) |
| CHU9D | 134 (80%) | 124 (74%) |
| EQ-5D-5L | 134 (80%) | 124 (74%) |
| **Overall** |  |  |
| Primary analysis: SDQ at 12-months | 128 (77%) | 133 (79%) |
| Secondary analysis: SDQ at 6-months | 136 (82%) | 143 (85%) |
| Secondary analysis: CHU9D at 12-months | 115 (69%) | 105 (63%) |
| Secondary analysis: EQ-5D-5L at 12-months | 114 (69%) | 106 (63%) |
| Secondary analysis: CHU9D + EQ-5D-5L at 12-months | 113 (68%) | 105 (63%) |

**Table S5: Hospital and community health and social care service use over the 12-month follow-up period**

| **Service** | **MICE**  **(N=128)** | | | | **Control**  **(N=133)** | | | |
| --- | --- | --- | --- | --- | --- | --- | --- | --- |
|  | **N** | **Mean (SD)** | **Range** | **% using** | **N** | **Mean (SD)** | **Range** | **% using** |
| **MICE intervention** |  |  |  |  |  |  |  |  |
| Total sessions | 128 | 18.23 (3.90) | 2 - 23 | 100% | n/a | n/a | n/a | n/a |
| Intervention sessions | 128 | 16.50 (3.65) | 2 - 21 | 100% | n/a | n/a | n/a | n/a |
| Booster sessions | 116 | 1.73 (0.62) | 0 - 2 | 90% | n/a | n/a | n/a | n/a |
| **Hospital services** |  |  |  |  |  |  |  |  |
| Inpatient – Epilepsy | 25 | 0.77 (2.00) | 0 - 10 | 20% | 28 | 0.82 (2.28) | 0 - 17 | 21% |
| Inpatient - Mental health | 1 | 0.01 (0.09) | 0 - 1 | 1% | 0 | 0.00 (0.00) | 0 | 0% |
| Inpatient – Other (not specified) | 17 | 0.41 (1.52) | 0 - 12 | 13% | 33 | 1.05 (3.49) | 0 - 22 | 25% |
| Outpatient – Epilepsy | 107 | 2.97 (3.50) | 0 - 28 | 84% | 113 | 3.82 (5.98) | 0 - 58 | 85% |
| Outpatient - Mental health | 15 | 0.21 (0.66) | 0 - 3 | 12% | 18 | 0.48 (2.02) | 0 - 18 | 14% |
| Outpatient – Other (not specified) | 87 | 3.06 (4.95) | 0 - 33 | 68% | 99 | 4.11 (6.48) | 0 - 42 | 74% |
| Accident & Emergency | 30 | 0.39 (0.86) | 0 - 5 | 23% | 33 | 0.44 (1.33) | 0 - 12 | 25% |
| Ambulance | 18 | 0.50 (1.55) | 0 - 9 | 14% | 37 | 0.67 (1.49) | 0 - 10 | 28% |
| **Community services** |  |  |  |  |  |  |  |  |
| General practitioner | 25 | 0.77 (2.00) | 0 - 10 | 20% | 28 | 0.82 (2.28) | 0 - 17 | 21% |
| Nurse/school nurse | 93 | 4.22 (8.63) | 0 - 71 | 73% | 96 | 5.18 (13.78) | 0 - 120 | 72% |
| Community paediatrician | 38 | 0.58 (1.19) | 0 - 9 | 30% | 31 | 0.51 (1.35) | 0 - 10 | 23% |
| Psychiatrist | 2 | 0.08 (0.63) | 0 - 6 | 2% | 14 | 0.41 (1.78) | 0 - 14 | 11% |
| CAMHS | 26 | 0.77 (2.34) | 0 - 13 | 20% | 24 | 0.86 (4.12) | 0 - 44 | 18% |
| Clinical psychologist | 13 | 0.4 (2.31) | 0 - 22 | 10% | 22 | 0.56 (1.87) | 0 - 12 | 17% |
| Counsellor | 4 | 0.30 (1.98) | 0 - 18 | 3% | 16 | 1.12 (4.29) | 0 - 30 | 12% |
| Education psychologist | 36 | 1.88 (6.31) | 0 - 43 | 28% | 40 | 1.88 (4.80) | 0 - 27 | 30% |
| Speech & language therapy | 32 | 4.38 (13.62) | 0 - 85 | 25% | 39 | 3.20 (8.65) | 0 - 56 | 29% |
| Physiotherapy | 31 | 2.11 (8.17) | 0 - 70 | 24% | 32 | 2.38 (8.10) | 0 - 58 | 24% |
| Occupational therapy | 32 | 4.56 (16.60) | 0 - 102 | 25% | 33 | 2.11 (7.28) | 0 - 56 | 25% |
| Art/drama/music/play therapy | 16 | 2.78 (10.61) | 0 - 85 | 13% | 20 | 2.83 (8.42) | 0 - 48 | 15% |
| Family therapy | 0 | 0.00 (0.00) | 0 | 0% | 2 | 0.39 (4.17) | 0 - 48 | 2% |
| Dietician | 13 | 0.25 (1.08) | 0 - 10 | 10% | 18 | 0.48 (1.68) | 0 - 15 | 14% |
| Social worker | 15 | 0.79 (3.79) | 0 - 30 | 12% | 21 | 1.50 (6.77) | 0 - 68 | 16% |
| Youth worker | 2 | 0.25 (2.05) | 0 - 20 | 2% | 6 | 0.55 (2.89) | 0 - 21 | 5% |
| Family support worker | 14 | 0.89 (4.01) | 0 - 26 | 11% | 11 | 0.56 (2.57) | 0 - 20 | 8% |
| Social inclusion worker | 2 | 0.02 (0.12) | 0 - 1 | 2% | 0 | 0.00 (0.00) | 0 | 0% |
| Foster care | 0 | 0.00 (0.00) | 0 | 0% | 0 | 0.00 (0.00) | 0 | 0% |
| Residential care | 0 | 0.00 (0.00) | 0 | 0% | 0 | 0.00 (0.00) | 0 | 0% |
| Respite care | 4 | 0.52 (4.15) | 0 - 45 | 3% | 7 | 0.56 (2.79) | 0 - 22 | 5% |

CAMHS=Child and Adolescent Mental Health Services

**Table S6: Prescribed medications over the 12-month follow-up period**

| **Medication for** | **Intervention**  **(N=128)** | | **Control**  **(N=133)** | |
| --- | --- | --- | --- | --- |
|  | **N** | **% using** | **N** | **% using** |
| Epilepsy | 123 | 96% | 122 | 92% |
| Mental health (anxiety, depression, ADHD) | 8 | 6% | 11 | 8% |
| Other | 62 | 48% | 61 | 46% |

ADHD=attention deficit hyperactivity disorder

**Figure S1: Cost-effectiveness plane for SDQ**


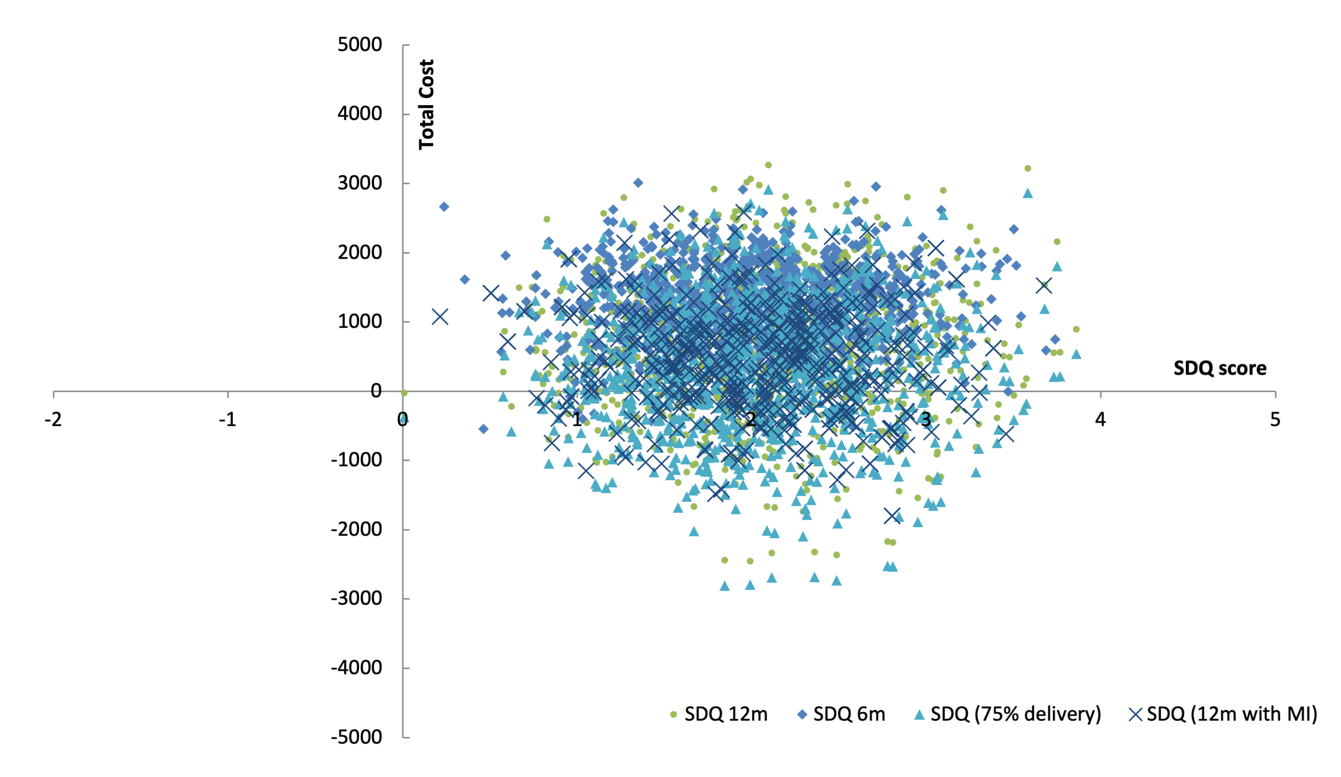


**Figure S2: Cost-effectiveness plane for QALYs**


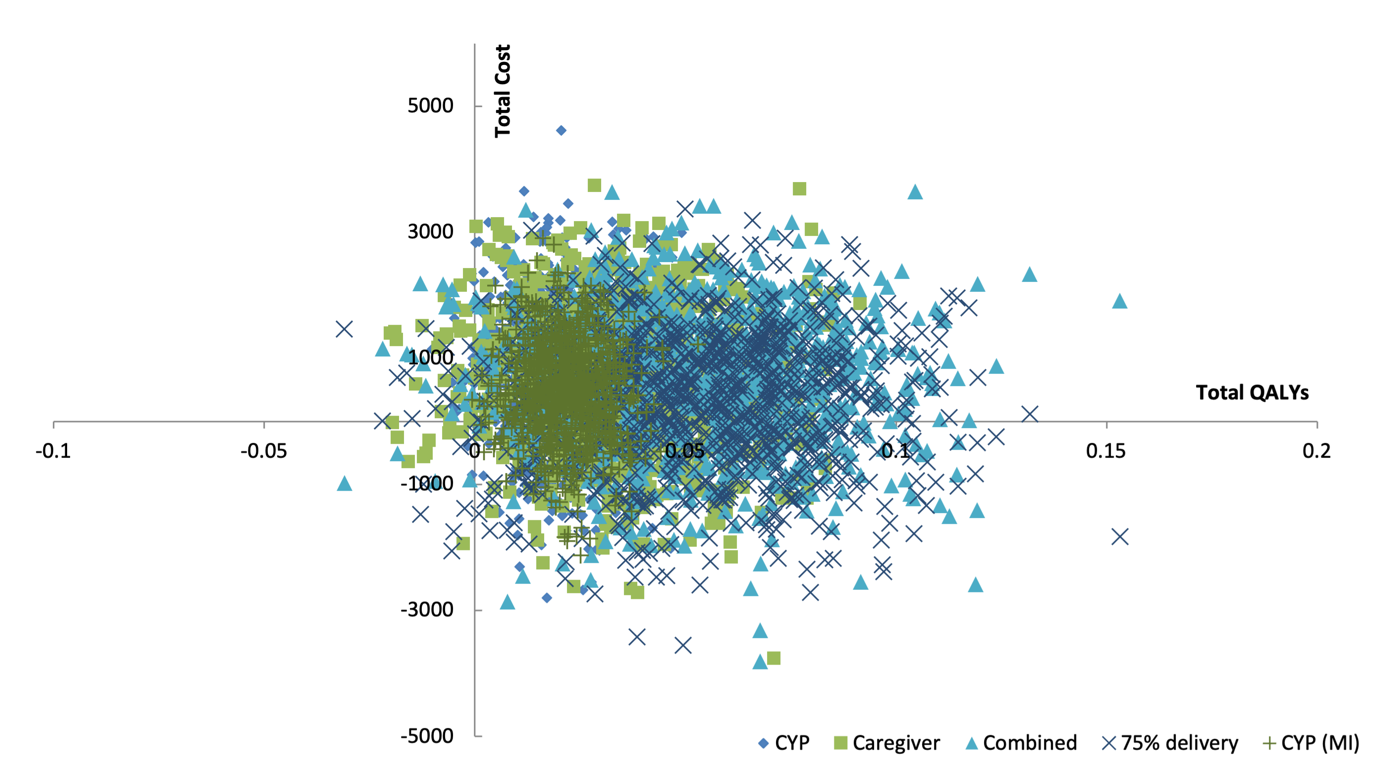


**References**

1. Department of Health (DH). NHS Reference Costs 2020-21. London DH, 2021.

2. Jones K, Burns A. Unit Costs of Health and Social Care 2021. Personal Social Services Research Unit: University of Kent, Canterbury, 2021.

3. Royal Pharmaceutical Society of Great Britain. British National Formulary for Children. 2021. <https://about.medicinescomplete.com/publication/british-national-formulary-for-children/>.

4. NHS Business Services Authority Statistics. Prescription Cost Analysis - England 2021. 2021. <https://www.nhsbsa.nhs.uk/statistical-collections/prescription-cost-analysis-england/prescription-cost-analysis-england-2020-21>.

5. Coughtrey AE, Bennett SD, Stanick C, et al. Training and supervision of physical health professionals to implement mental health care in paediatric epilepsy clinics. *Epilepsy & Behavior* 2024; **157**: 109905.
